# Does Enhancing Paid Maternity Leave Policy Help Promote Gender Equality? Evidence from 31 Low- and Middle-Income Countries

**DOI:** 10.1007/s12147-021-09293-4

**Published:** 2021-12-21

**Authors:** Yan Chai, Vanessa Ríos-Salas, Pam Stek, Jody Heymann

**Affiliations:** WORLD Policy Analysis Center, Fielding School of Public Health, University of California, 621 Charles E. Young Drive South, Los Angeles, CA 90095 USA

**Keywords:** Paid maternity leave, Gender equality, Gender norms, Workplace equality, Difference-in-differences

## Abstract

Globally, women continue to have less economic decision-making power and face gender-unequal norms at work. Little is known about the impact of national public policies on norms surrounding equality. We examined the impact of extending paid maternity leave policy on decision making in the household and gender norms in the workplace, specifically whether women have sole or joint decision-making power with respect to large household purchases and whether women are perceived as having an equal right to jobs when jobs are scarce. We used difference-in-differences models to analyze the impact of increasing paid maternity leave on outcomes measured in the Demographic Health Surveys and World Values Surveys collected in 31 low- and middle-income countries. A one-month increase in the legislated duration of paid maternity leave increased the odds that women and their partners/spouses reported that women had more decision-making power by 40% (95% CI 1.14, 1.70) and 66% (95% CI 1.36, 2.03), respectively. A one-month increase in the legislated duration of paid maternity leave was associated with 41.5 percentage-point increase in the prevalence of individuals disagreeing with the statement that “when jobs are scarce, men should have more right to a job than women.” More generous maternity leave increases gender equality in economic decision making in the household and improves gender norms related to work. Future studies should examine the impact of paternity leave and non-discrimination policy, as well as other large-scale policies aiming to improve gender equality at work and at home.

## Introduction

Gender norms influence women’s and girl’s opportunities and outcomes in a range of spheres including education. In households where mothers have limited decision-making authority, parents tend to have lower educational aspirations for their daughters, and girls’ school attendance rates lag those of their male peers [17, 39, 46, 49]. A study in the United States found that young adults who supported women’s equality in the workplace and women’s decision-making authority in the home spent more time in school and were more likely to earn a degree than their peers who held less egalitarian gender attitudes [16]. In addition, gender stereotypes that perpetuate occupational segregation and assign aptitude and ability on the basis of gender can shape girls’ educational choices, pushing them away from disciplines such as mathematics and science [9]. However, studies also demonstrate that modifying gender norms can change educational outcomes. For example, a study in India of a secondary school program that utilized classroom discussions to dispel gender stereotypes and endorse greater gender equality at home and in the workplace found that student participants adopted more egalitarian gender beliefs. In addition, evidence from the study suggests that the program increased girls’ intentions to attend college [19].

Gender norms also influence women’s patterns of work, inside and outside the home. Research has shown that attitudes about women’s roles in the workplace and their decision-making autonomy in the household are associated with the amount of time men and women spend on housework and the types of tasks they perform [1, 13]. In countries with more egalitarian gender norms, men devote more time to total work (including unpaid work), reducing the gender gap in housework [12, 23]. In addition, gender norms are a key determinant of women’s labor force participation rates, earnings, and promotional opportunities. In countries around the world, studies have found that gender ideologies that prioritize jobs for men and uphold the gendered segregation of labor are associated with lower rates of women in the paid workforce and greater gender pay gaps [14, 18, 25, 29]. Evidence from a study of men and women business leaders in Europe and the United States suggests that women are less likely than their male counterparts to be judged as effective leaders and problem solvers, stereotypes that diminish women’s opportunities for advancement [44]. Gender attitudes also influence agricultural production. In Burkina Faso, where household members generally gain access to land through the male household head, fertilizer is disproportionately concentrated on plots cultivated by men; all else being equal, men’s authority to make resource allocation decisions leads to lower yields on women’s plots [56]. Similarly, evidence from Kenya shows that women maize farmers’ empowerment, a measure defined in part by the level of their decision-making authority, is associated with increased maize productivity [20].

Studies show that disrupting inegalitarian gender norms can empower women economically and in the home. In Saudi Arabia, three-quarters of married men surveyed underestimated the level of their peers’ support for women working outside the home. Correcting husbands’ views of their communities’ acceptance of women’s employment resulted in their wives being more likely to apply and interview for jobs outside the home [10]. A study in Malawi found that an intervention promoting gender equality in the division of labor and in decision-making led to a significant shift toward the sharing of agricultural and household tasks and greater agreement on household expenditures [24].

Gender attitudes are also associated with a number of women’s health decisions and outcomes, including prenatal and obstetric care utilization [40], use of birth control [53], cervical cancer screening rates [28], parents’ care-seeking behaviors for their children [4, 55], and excess mortality of post-reproductive age women [11]. In addition, inequitable gender norms result in the prioritization of men’s health services, medical research, and physician education, all of which diminish women’s quality of care. Gender stereotypes that posit men as “curers” and women as “carers” segregate women into lower-paid health care occupations and reduce the share of female physicians, an outcome associated with lower health measures for women and their families [32]. Again, however, interventions that change gender attitudes can impact women’s health decisions and access to health care. A gender equity program in India undertaken to improve married women’s sexual health led community leaders and men to adopt significantly more egalitarian gender beliefs [50]. Other studies have shown that programs designed to transform inequitable gender norms can improve women’s reproductive health outcomes [38].

Another aspect of well-being influenced by gender attitudes is women’s vulnerability to gender-based violence. Studies show that gender norms are an important determinant of men’s propensity to perpetrate intimate partner violence (IPV). When women have less household decision-making authority and are more financially dependent on male partners, they are at greater risk, first, of becoming victims of IPV and, second, of lacking resources to improve their situation [30, 51, 57]. Programs that address inequitable gender norms can impact men’s use of violence against women [6]. For example, in Côte d’Ivoire and Ethiopia, interventions that incorporated gender dialogue and education groups and addressed gendered social inequalities significantly reduced the incidence of IPV [31, 45].

Another critical domain influenced by gender norms is political representation. Studies have shown that gender attitudes shape voters’ perceptions of women candidates’ competence and integrity, factors that influence women’s electoral success [8, 21, 26]. In Italy, mayors of municipalities who fail to consolidate support for their policies can be forced out of office by assembly members. A study there found that female mayors were more likely than their male counterparts to be ousted before the end of their terms by municipal councils' no-confidence votes and that unfavorable regional attitudes toward working women represented a significant predictor of women’s shortened mayoral tenures [27]. In Oman, beliefs about women’s role in society, including women’s equality in the workplace, strongly determine support for women’s political representation [3]. Similarly, in their study of forty-six countries worldwide, Paxton and Kunovich found that countries with more egalitarian gender attitudes, including support for working women, have higher levels of women’s political representation and that gender ideologies are the strongest predictors of differences in women’s political representation across countries [43].

Taken together, the existing evidence demonstrates the significant impact of gender norms on women’s educational, economic, health, and leadership outcomes. Studies also show that gender norms are not static and that changes in gender ideologies can lead to improvements in women’s opportunities and status. However, the question of how to alter restrictive, entrenched gender stereotypes remains.

Our understanding of how policy change can shift gender norms in particular is nascent, but there is strong evidence that legislative change can influence social attitudes in other areas. Studies have shown that social norms can shift in response to policy and legislative changes on smoking bans [42], welfare reform [33], criminal penalties for domestic violence [48], and immigration law [47]. In addition, although the research is limited, a small number of studies have established causality between policy changes and transformations in gender ideologies. For example, reserving village council spots for women in India weakens gender stereotypes of women’s roles and improves perceptions of women as effective leaders, thereby expanding women’s access to public office [7]. Research has also shown that legal change can shift attitudes toward sexual minorities and same-sex marriage [2, 22, 37, 54].

In this study, we look at an important example of whether laws can shift norms about women’s role in the labor force and in the household. The question we examine is whether policy changes meant to improve women’s and their families’ ability to balance work and caregiving roles can reduce gender inequalities in norms. Specifically, we analyze the impact of legislated paid maternity leaves on household norms of decision making and norms about gender equal participation in the labor force.

Policy feedback theory suggests that changes in paid maternity leave benefits represent a potentially fruitful field of study with respect to policy change impact on community gender norms. Soss and Schram [52] propose a policy feedback analysis framework based on the two dimensions of visibility and proximity. In this model, policies that have greater salience to and a greater direct impact on large swathes of the population are more likely to produce feedback, including changes in attitudes about the policies and associated issues. Paid maternity leave benefits are both proximate and visible in low- and middle-income countries. Sixty-four percent of all workers in the countries studied were aged between 20 and 44 in 2019; thus, more than 6 in 10 workers would potentially be impacted by a change in paid maternity leave benefits for themselves or their partners [36]. Women’s paid labor is also highly visible. In 2019, the labor force participation rate of women aged 15–64 stood at 50% for all low- and middle-income countries combined [59], and boosting female labor force participation rates is widely recognized as an important component of economic growth and the achievement of the UN Sustainable Development Goals. These twin attributes of high visibility and high proximity suggest that changes in paid maternity leave policies have a strong potential to impact gender norms.

## Methods

### Data Sources

Longitudinal data measuring national maternity leave policies for each United Nations (UN) member state were made available by the University of California Los Angeles’ WORLD Policy Analysis Center and then collected retrospectively to 1995 for countries with comparable longitudinal and household survey data by McGill University’s Policy-Relevant Observational Studies for Population Health Equity and Responsible Development (PROSPERED) project [5]. In order to obtain information on maternity leave protections, our research team conducted a comprehensive review of all national labor legislation collected by the International Labour Organization (ILO), and available in their NATLEX online database in original languages and in translations. Information was standardized by our research team into quantitatively comparable indicators. All materials were coded independently by two researchers and compared to ensure accuracy. Additional quality controls were performed upon completion. Further details regarding the collection and coding of global maternity leave policies are available elsewhere [34].

For the analysis of decision making in the household, we obtained individual-level data from the Demographic Health Surveys (DHS), nationally representative cross-sectional household surveys collected since the mid-1990s in LMICs. The DHS collect information about participation in household decision making for women of reproductive age (15–49 years), as well as reproductive and child health. In some surveys, men ages 15–54 years are also eligible to participate; depending on the country, they are sampled similarly to women or they constitute a sub-sample of either one-third or 50% of households selected for the survey [15].

Questions on attitudes regarding women’s work equality were collected from the World Values Survey (WVS). The WVS, covering nearly 90% of the global population, are a collection of nationally representative time-series data obtained from almost 100 countries. Trained interviewers and structured questionnaires are used to ask selected individuals, in their local language, questions regarding their beliefs, values, and motivations, including women’s status and gender roles. The minimum number of completed interviews for most countries is 1200, and individuals are selected through either a full probability or a combination of probability and stratified sampling methods.

### Exposure Variable

The exposure of interest in our study was the legislated length of paid maternity leave for each sampled country between 1995 and 2016. We recorded the legislated length of paid leave available through national maternity leave policies for which only women are eligible. To ensure temporality associated with causality, as well as reduce exposure misclassification, observations in each survey were assigned the legislated length of paid maternity leave one year prior to the survey year. We did not distinguish between leave that could be taken before birth and leave that could be taken after birth.

### Analysis of Household Decision Making

#### Outcome Variable

To capture changes in attitudes toward decision making in the household, we used women’s economic decision-making power as reported by women and their male partners/spouses. Our analysis focuses on women’s decision-making authority with respect to major household purchases, information which is available for both women and their partners/spouses. In most DHS of phases 5–7, women and their partners/spouses were asked independently: “Who usually makes decisions about making major household purchases?” Response options included respondent, husband (wife)/partner, respondent and husband (wife)/partner jointly, someone else, and other. The wording of the questions was slightly different in phase 4 and some surveys in later phases (e.g., Colombia 2015–2016): “Who in your family usually has the final say on the following decision: making large household purchases?” Response options included respondent, husband (wife)/partner, respondent and husband (wife)/partner jointly, someone else, respondent and someone else jointly, and decision not made or not applicable. Our measures for women’s decision-making assessed responses by female partners/spouses and male partners/spouses, coded as 1 if women were reported as having sole or joint decision-making power in each case; otherwise, the variable took a value of 0.

#### Sample

Our empirical analysis of decision making in the household was restricted to DHS from the fourth round onward because they include information on women’s participation in household decision making, our proxy for gender norms. We considered only those DHS that asked women and their partners or spouses independently about women’s decision-making authority in their households. Our final sample comprised 19 countries (Table 1), seven of which experienced a change in the duration of paid maternity leave between 1995 and their latest year of survey available (“treatment group”). The rest of the countries did not experience a change in the duration of paid maternity leave policy (“control group”). Given our measure of maternity leave—the duration of the leave in the previous year—available starting in 1996, we considered only women who gave birth to their last child between 1996 and 2016 (or latest survey available) from the surveys, as we used the birth year of the last child as a proxy to define the mothers that were affected (post-policy) and not affected (pre-policy) by the change in the duration of maternity leave. Our final sample included 101,982 married couples where women were between 15 and 49 years old. We restricted the sample to couples who reported being married or living together as married because our proxy of gender norms is available only for married men in some surveys. We then excluded couples who had missing data in our proxy of gender norms (1.2%), education attained (0.7%), and an indicator of other women in the household (0.5%), which are critical control variables in the analysis. After this, our base sample consisted of 100,294 couples.

**Table 1:** Sample description for the analysis of decision-making in the household by country

Country	Effective year of paid maternity leave extension*	Weeks of paid maternity leave before and after extension	Survey years of DHS used	Couples not exposed to a change in the duration of maternity leave*	Couples exposed to a change in the duration of maternity leave*	Total sample of married couples
Colombia	2011	11, 14	2015	2662	853	3515
Malawi	2000	0, 8	2000, 2015	1299	3454	4753
Myanmar	2012	12, 14	2015	1365	936	2301
Sierra Leone	2002	0, 12	2013	170	3191	3361
Uganda	2006	4.3, 12	2000, 2011, 2016	942	2406	3348
Zambia	2002, 2006	12, 12.8, 17.1	2013	292	6500	6792
Zimbabwe	2006	12.9, 14	2010, 2015	1140	4771	5911
All treated countries^a^			7870	22,111	29,981	
Burkina Faso	–	14	2003, 2010	6550	0	6550
Burundi	–	12	2010, 2016	4359	0	4359
Ethiopia	–	12.9	2011, 2016	11,340	0	11,340
Ghana	–	12	2003, 2014	3241	0	3241
Indonesia	–	12.9	2007, 2012	12,931	0	12,931
Mali	–	14	2001, 2012	4484	0	4484
Nepal	–	7.4	2000, 2010, 2016	5001	0	5001
Nigeria	–	12	2013	7698	0	7698
Rwanda	–	12	2010, 2014	3947	0	3947
Senegal	–	14	2010, 2014	6537	0	6537
Tanzania	–	12	2005, 2009, 2015	2225	0	2225
Togo	–	14	2013	2000	0	2000
All control countries ^b^			70,313	0	70,313	

In the empirical analyses, we assume a one-year lag between policy and its implementation

^*^We refer to changes in the duration of paid matertniy leave between 1995 and the most recent year of data available in the country

^a^Treated countries are countries that experienced a change in the duration of paid maternity leave between 1995 and 2016

^b^Control countries are countries that did not experience a change in the duration of paid maternity leave between 1995 and 2016

#### Control Variables

For the analysis of decision making in the household, we adjusted for a wide set of individual- and country-level characteristics, as summarized in Appendix Table 9. Individual-level characteristics include women’s age, education, relation to household’s head, difference with partners’ or spouses’ age, difference with partners’ or spouses’ education, number of children, area of residence, household wealth index, and number of other women in the household. Our fully adjusted model also included country-level characteristics that may influence paid maternity leave policy reforms and be associated with changes in attitudes toward women’s economic decision-making power in the household. Gross Domestic Product (GDP) per capita based on purchasing power parity was extracted from the World Bank’s World Development Indicators and Global Development Finance databases.

#### Effect of Paid Maternity Leave Policy on Household Decision Making

To estimate the influence of change in duration of paid maternity leave policy on decision making in the household, we estimated the following equation:

$$\log {\text{it}}\left( {{h_{{\text{icb}}}}} \right) = {\beta_0} + {\beta_1}{\text{ma}}{{\text{t}}_{{\text{cb}}}} + {X_{{\text{ict}}}}{\beta_2} + \;{\beta_3}{Z_{{\text{ct}}}} + {\lambda_c} + \;{\gamma_b} + {\delta_t} + {\varepsilon_{{\text{icb}}}}$$

where ${h_{{\text{icb}}}}$ represents the decision-making authority of woman $i$, whose last child was born in country $c$ in year $b$. The main independent variable, ${\text{ma}}{{\text{t}}_{{\text{cb}}}}$ is the duration of maternity leave. ${X_{{\text{ict}}}}$ represents a set of individual-specific covariates that are expected to influence decision making in the household. These include women’s and their partners’ or spouses’ basic characteristics and their household wealth, among other variables (see Appendix Table 9 for a more detailed description of these variables and their measurement). ${Z_{{\text{ct}}}}$ is a set of country-specific, time-varying covariates measured for the year in which women and their partners or spouses were surveyed. We control for another policy that is related to maternity leave and that might affect norms: the availability of paid paternity leave (see Appendix Table 9). The models also include country-specific fixed effects (${\lambda_c}$) to control for time-invariant differences across countries, fixed effects of the last child’s year of birth (${\gamma_b})$, and fixed effects to control for shocks in any survey year (${\delta_t}$). The equations were estimated using logistic regressions, because our measures of gender norm are binary variables. All regressions were weighted taking into account the DHS design [35]. Therefore, the standard errors were clustered by the primary sample units in each country-survey. Sampling weights were rescaled to assign equivalent importance to each country because not all countries had the same number of surveys, and we did not want the population size of a country to affect the overall results.

### Analysis of Gender Norms at Work

#### Outcome Variable

To capture changes in attitudes toward women’s equal rights to employment, we utilized responses to a WVS question that was asked of both women and men in every wave across our time periods of interest (1990–2015). The question asked individuals to respond to the following statement: “When jobs are scarce, men should have more right to a job than women.” Possible responses were “agree,” “disagree,” “I don’t know,” and “neither.” In the analysis, those who selected the response “I don’t know” or “neither” were grouped together and coded as “neither.”

#### Sample

For the analysis of women’s equal rights to employment our sample comprised 53,811 individuals between ages 15 and 60 (inclusive) from 41 WVS across 17 lower-middle-income and low-income countries (Table 2). These 17 countries were selected based on the availability of at least two WVS administered between 1990 and 2015, which allowed for the utilization of the difference-in-differences approach. Treated and control countries were distinguished based on whether or not they experienced a change in national paid maternity leave policy. The three treated countries (i) experienced at least one change in the duration of paid maternity leave policy between 1995 and 2016 and (ii) had at least one WVS before and after the policy change.

**Table 2:** Sample description for the analysis of gender equality in the workplace by country

Country	Effective year of paid maternity leave extension*	Weeks of paid maternity leave before and after extension	Survey years of WVS used	Total sample size
Georgia	2014	18, 26	2009, 2014	2125
Morocco	2004	12, 14	2001, 2007, 2011	3289
Zimbabwe	2006	12.9, 14	2001, 2012	2333
All treated countries^a^				7747
Armenia	–	20	1997, 2011	2379
Bangladesh	–	12	1996, 2002	2262
Egypt	–	13	2001, 2008, 2012	6693
Ghana	–	12	2007, 2012	2910
India	–	12	2001, 2006, 2012	6145
Indonesia	–	12.9	2001, 2006	2789
Jordan	–	10	2001, 2014	2176
Nigeria	–	12	2000, 2012	3620
Pakistan	–	12	1997, 2001, 2012	3704
Philippines	–	9	1996, 2001, 2012	3210
Republic of Moldova	–	18	1996, 2002, 2006	2474
Rwanda	–	12	2007, 2012	2433
Ukraine	–	18	1996, 2006, 2011	3944
Vietnam	–	17	2001, 2006	1325
All control countries ^b^				46,064

In the empirical analyses, we assume a one-year lag between policy and its implementation

^*^We refer to changes in the duration of paid maternity leave between 1995 and the most recent year of data available in the country

^a^Treated countries are countries that experienced a change in the duration of paid maternity leave between 1995 and 2016

^b^Control countries are countries that did not experience a change in the duration of paid maternity leave between 1995 and 2016

#### Control Variables

For the analysis of women’s equal rights to employment, we identified potential confounders and other determinants of attitudes regarding women’s work equality in LMICs based on a literature review (Appendix Table 10). Individual-level characteristics included sex, age (numerical), year of birth, marital status (i.e., married or living together as married, divorced, separated, or widowed, single or never married), current work status (i.e., working, not working), education (i.e., none or incomplete primary, completed primary, incomplete secondary, completed secondary, some university or more). Our fully adjusted model also included country-level characteristics that may influence paid maternity leave policy reforms and be associated with changes in attitudes toward women’s work equality. GDP per capita based on purchasing power parity, labor force participation among women aged 15 to 64, and unemployment as a percent of the female labor force were extracted from the World Bank’s World Development Indicators and Global Development Finance databases.

#### Effect of Paid Maternity Leave Policy on Gender Norms at Work

We estimated the effect of a one-month increase in paid maternity leave policy on the prevalence of not agreeing (i.e., “disagree” or “don’t know/neither”) with the statement “When jobs are scarce, men should have more right to a job than women” using the following multinomial logistic regression models:

$$\ln \left( {\frac{{P\left( {{Y_{ijt}} = {\text{agree}}} \right)}}{{P\left( {{Y_{ijt}} = {\text{disagree}}} \right)}}} \right) = {\beta_{10}} + {\beta_{11}} * {M_{jt - 1}} + \sum {\beta_{1n}} * {Z_{ijt}} + \sum {\beta_{1k}} * {C_{jt - 1}} + {\lambda_j} + {\delta_t}$$

$$ln\left( {\frac{{P\left( {{Y_{ijt}} = agree} \right)}}{{P\left( {{Y_{ijt}} = neither} \right)}}} \right) = {\beta_{20}} + {\beta_{21}}*{M_{jt - 1}} + \sum {\beta_{2n}}*{Z_{ijt}} + \sum {\beta_{2k}}*{C_{jt - 1}} + {\lambda_j} + {\delta_t}$$

where ${Y_{ijt}}$ represents the outcome (i.e., agree, disagree or neither agree or disagree with the statement “When jobs are scarce, men should have more right to a job than women”) for an individual *i* surveyed in country *j* in year *t,* and ${M_{jt - 1}}$ is the calculated months of paid maternity leave in country *j* one year before the survey year (*t* – 1). Vector ${Z_{ijt}}$ represents the individual-level characteristics that we adjusted in the model. We also controlled for time-varying, country-level confounders measured one year before the survey year (*t* – 1), represented by the vector ${C_{jt - 1}}$. Fixed effects for country (${\lambda_j}$) and year (${\delta_t}$) were included to account for, respectively, unobserved time-invariant confounders that vary across countries and temporal trends in the outcome shared across countries. Average marginal effects were calculated from the multinomial logistic regression models to obtain estimates on the additive scale. All models incorporated respondent-level sampling weights to account for individual survey sampling designs and cluster-robust standard errors to account for clustering at the country level. Statistical analyses were performed using Stata software version 16 (Stata Corp, College Station, TX).

### Temporality

For both analyses of decision-making in the household and gender equality in the workplace, sensitivity analyses using policy with different lead times, specifically the length of paid maternity leave one, two, and three years after the survey year (*t* + 1, *t* + 2, *t* + 3), were used to examine the robustness of our main estimates. Analyses with lead times were used to test whether policy effects could be detected before the actual year of policy implementation, which would be inconsistent with the inference that paid maternity leave had a causal effect on gender norms.

### Examination of Parallel Trends Assumption

One of the primary assumptions in the difference-in-differences approach is the parallel trends assumption [58]. That is, in the absence of treatment, trends in outcomes between treated and control groups remain the same over time. For the analysis of decision-making in the household, we examined the parallel trends assumption by comparing the prevalence of the respondent, separately for women and their partner or spouse, reporting women have sole or joint decision-making authority in major household purchases among treated and control groups. For the analysis of gender equality in the workplace, we compared the prevalence of the respondent not agreeing with the statement “when jobs are scarce, men should have more right to a job than women” among treated and control groups.

## Results

### Descriptive Statistics

Between 1995 and 2016, the average length of paid maternity leaves among the 22 countries that did not change the duration of leave available was 13.19 weeks. Among the nine countries (i.e., Colombia, Georgia, Malawi, Morocco, Myanmar, Sierra Leone, Uganda, Zambia, Zimbabwe) that changed the duration of leave available, paid maternity leave increased on average from 9.13 to 14.57 weeks between 1995 and 2016 (Tables 1 and 2).

In the study sample of decision making in the household, women were 31 years old on average while men were 38 years old on average. Over 38% of women had no education, about 21% of women had some primary education, 12% of women completed primary education only, 16% had some secondary education, and only 12% of women completed secondary education or higher. Half of the women in the sample had a partner or spouse at the same education level and one third of women had a partner or spouse with education level higher than hers. Twenty-six percent of the women in the sample lived with a partner or spouse 3 to 5 years older than her and half of the women in the sample lived with a partner or spouse at least 6 years older than her (Table 3).

**Table 3:** Characteristics of the study sample for the analysis of household decision making, *N* = 100, 294

Average women’s age (SD)	31.46 (0.02)
Average men’s age (SD)	37.91 (0.03)
Women’s education	
No education	38,947 (38.83%)
Incomplete primary	21,097 (21.04%)
Complete primary	12,435 (12.4%)
Incomplete secondary	15,897 (15.85%)
Complete secondary	6878 (6.86%)
Higher	5040 (5.03%)
Difference in age	
Wife is older than partner or spouse	6148 (6.13%)
Wife is the same age as partner or spouse	4128 (4.12%)
Partner or spouse is 1 or 2 years older	13,618 (13.58%)
Partner or spouse is 3 to 5 years older	26,200 (26.12%)
Partner or spouse is 6 to 10 years older	25,467 (25.39%)
Partner or spouse is more than 10 years older	24,733 (24.66%)
Difference in education	
Wife’s education is higher than partner’s or spouse’s education	15,125 (15.08%)
Their education level is the same	51,492 (51.34%)
Partner’s or spouse’s education is higher than wife’s education	33,677 (33.58%)

In the study sample of gender norms at work, both men and women were 35 years old on average. Sixty-nine percent of the women in the sample were married or living together as married and 21% of the women in the sample reported single or never married. Sixty-five percent of men reported being married or living together as married; 31% of men in the sample were single or never married. Nearly 24% of the women in the sample did not complete primary education while only 17% of men reported the same. 10% of the men and 11% of the women in the sample completed primary education only and 52% of the women completed secondary education or higher while 58% of the men reported the same (Table 4).

**Table 4:** Characteristics of the study sample for the analysis of gender norms at work, *N* = 53,811

Sex	
Male	25,877 (48.09%)
Female	27,934 (51.91%)
Average women’s age (SD)	35.13 (0.69)
Average men’s age (SD)	35.48 (0.60)
Women’s marital status	
Married or living together as married	19,364 (69.32%)
Divorced/separated/widowed	2646 (9.47%)
Single/never married	5924 (21.21%)
Men’s marital status	
Married or living together as married	16,942 (65.47%)
Divorced/separated/widowed	700 (2.71%)
Single/never married	8235 (31.82%)
Women’s education	
None or incomplete primary	6684 (23.93%)
Completed primary	3006 (10.76%)
Incomplete secondary	3569 (12.78%)
Completed secondary	9546 (34.17%)
Some university or more	5129 (18.36%)
Men’s education	
None or incomplete primary	4277 (16.53%)
Completed primary	2649 (10.24%)
Incomplete secondary	3795 (14.67%)
Completed secondary	9357 (36.16%)
Some university or more	5799 (22.41%)

### Examination of Parallel Trends Assumption

**Fig. 1:**
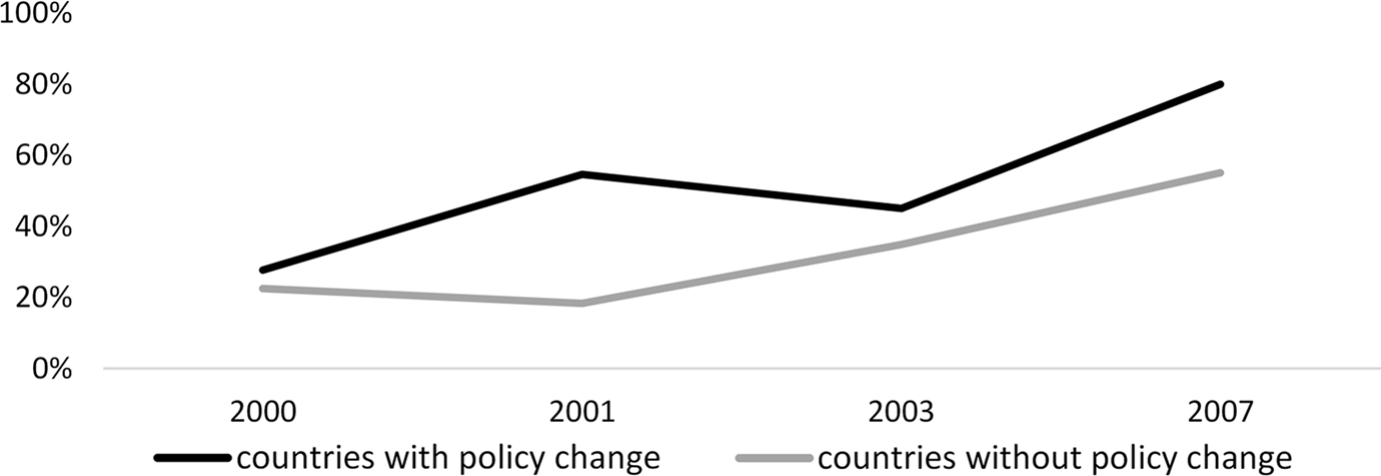
Trends in average proportion of women reported having sole or joint decision-making authority in major household purchase in control and treated countries in the pre-intervention period, 2000–2007

**Fig. 2:**
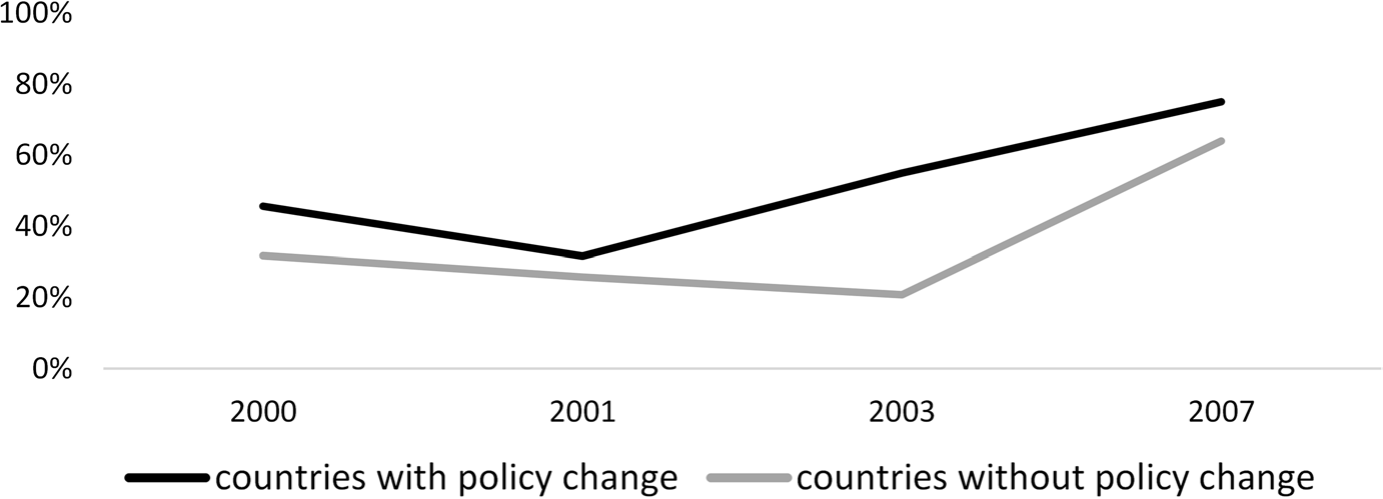
Trends in average proportion of men reported women having sole or joint decision-making authority in major household purchases in control and treated countries in the pre-intervention period, 2000–2007

**Fig. 3:**
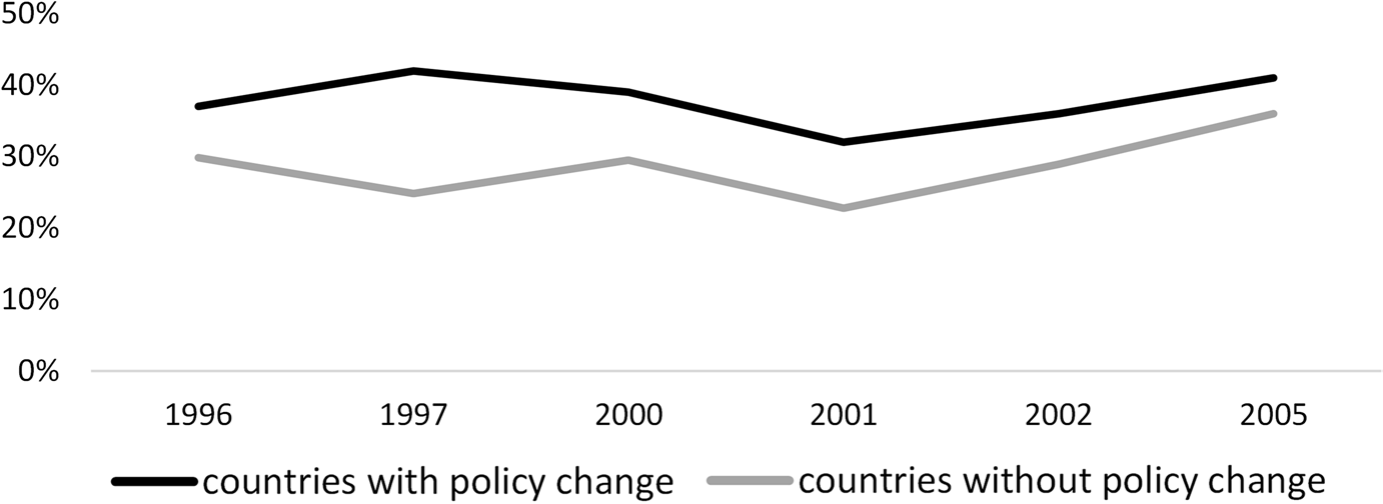
Trends in average proportion of individual disagreeing with the statement in control and treated countries in the pre-intervention period, 1996–2005

For the analysis of decision-making in the household, the trends in the prevalence of women and their partner or spouse reporting women having sole or joint decision-making authority in major household purchases were similar among treated and control countries (Fig. 1–2). For the analysis of gender equality in the workplace, the trends in the prevalence of the respondent not agreeing with the statement “when jobs are scarce, men should have more right to a job than women” were similar among treated and control countries (Fig. 3). These figures provided some evidence that the assumption was not violated. Limitations include that the parallel trends assumption is difficult to check visually in the generalized fixed-effects difference-in-differences design, with multiple countries with policy changes at multiple time points [58]. We lacked longitudinal measurements on our outcome for all sampled countries, as some countries had only one survey available before the policy reform.

### Effect of Paid Maternity Leave Policy on Economic Decision Making

The results of the logistic regressions of paid maternity leave on economic decision making are presented in Panel A of Table 5. The results show that a one-month increase of the legislated duration of paid maternity leave policy increased the odds that women had sole or joint decision-making authority in major household purchases by 40% (95% CI 1.14, 1.70) as reported by women and by 66% (95% CI 1.36, 2.03) as reported by their partners or spouses.

**Table 5:** Effect of an increase in length of paid maternity leave policy on the odds of sole or joint decision-making authority in major household purchases for women, *N* = 100,294

	Women	Men
	OR (95% CI)	OR (95% CI)
Panel A: Continuous measure of the duration of maternity leave		
1-month increase in length of paid maternity leave policy	1.40 (1.14, 1.70)	1.66 (1.36, 2.03)
Panel B: Categorical measure of the duration of maternity leave		
0–7 weeks of paid maternity leave policy	Ref	Ref
8–13 weeks of paid maternity leave policy	1.47 (1.22, 1.77)	1.77 (1.46, 2.15)
14–30 weeks of paid maternity leave policy	1.67 (1.30, 2.16)	1.84 (1.42, 2.39)

All regressions are weighted and adjusted for country, the last child’s year of birth, and survey year fixed effects, and a set of control variables as described in Appendix Table 9

To test a non-linear association between paid maternity leave policy and economic decision making, we ran similar regressions to those presented in Panel A using a categorical measure of the duration of paid maternity leave policy. Results in Panel B show that living in a country with eight or more weeks of paid maternity leave had a positive influence on decision-making authority in the household. Women living in countries with 14 to 30 weeks of paid leave (the maximum paid leave available in our sample) had higher odds of having decision-making authority in the household than their peers with only 8 to 13 weeks of leave available.

### Effect of Paid Maternity Leave Policy on Perceived Right to Work

Table 6 Panel A shows the effect of a one-month increase in the length of paid maternity leave on the change in prevalence of individuals not agreeing with the statement “When jobs are scarce, men should have more right to a job than women” in lower-middle-income and low-income countries. The result indicated that a one-month increase in the legislated duration of paid maternity leave was associated with 41.5 percentage-point increase in the prevalence of individuals disagreeing with the statement.

**Table 6:** Effect of a 1-month increase in length of paid maternity leave policy on the prevalence of individuals disagreeing with the statement “when jobs are scarce, men should have more right to a job than women”, in different subpopulations

	Disagree
Panel A: in lower middle and low income countries, *N* = 53,811	
1-month increase in length of paid maternity leave policy	41.5 (20.3, 62.6)
Panel B: lower middle and low income countries, men only, *N* = 25,877	
1-month increase in length of paid maternity leave policy	28.4 (8.1, 48.8)
Panel C: lower middle and low income countries, women only, *N* = 27,934	
1-month increase in length of paid maternity leave policy	54.1 (31.7, 76.5)

All regressions are weighted and adjusted for country and survey year fixed effects, and a set of control variables as described in Appendix Table 10

Reported estimates are marginal effects, which were multiplied by 100 in order to be interpreted as the percentage point difference in prevalence

95% confidence intervals are in parentheses

Table 6 Panel B shows the effect of a one-month increase in the length of paid maternity leave on the change in prevalence of not agreeing with the statement “When jobs are scarce, men should have more right to a job than women” among only men in lower-middle-income and low-income countries. The result indicated that a one-month increase in the legislated duration of paid maternity leave was associated with 28.4 percentage-point increase in the prevalence of disagreeing with the statement among men.

Table 6 Panel C shows the effect of a one-month increase in the length of paid maternity leave on the change in the prevalence of not agreeing with the statement “When jobs are scarce, men should have more right to a job than women” among only women in lower-middle-income and low-income countries. The result indicated that a one-month increase in the legislated duration of paid maternity leave was associated with 54.1 percentage-point increase in the prevalence of disagreeing with the statement among women.

### Temporality

The results of these sensitivity analyses support the temporality between changes in paid maternity leave policy and the household decision-making outcome and workplace gender equality outcome (Tables 7, 8).

**Table 7:** Sensitivity analyses of the effect of a 1-month increase in length of paid maternity leave policy on the odds of sole or joint decision-making authority in major household purchases for women, with different lead times on policy

	Women	Men
	OR (95% CI)	OR (95% CI)
1-month increase in length of paid maternity leave policy		
Lead one year, t + 1	1.02 (0.78, 1.33)	0.91 (0.71, 1.17)
Lead two years, t + 2	1.04 (0.82, 1.31)	0.97 (0.78, 1.21)
Lead three years, t + 3	1.11 (0.85, 1.44)	0.86 (0.68, 1.09)

Notes: All regressions are weighted and adjusted for country, the last child’s year of birth, and survey year fixed effects, and a set of control variables as described in Appendix Table 9

**Table 8:** Sensitivity analyses of the effect of a 1-month increase in length of paid maternity leave policy on the prevalence of individuals disagreeing with the statement “when jobs are scarce, men should have more right to a job than women”, with different lead times on policy

	Disagree
1-month increase in length of paid maternity leave policy	
Lead one year, t + 1	4.1 (-1.0, 9.3)
Lead two years, t + 2	4.9 (-1.1, 11)
Lead three years, t + 3	5.7 (-1.9, 13.3)

Notes: All regressions are weighted and adjusted for country and survey year fixed effects, and a set of control variables as described in Appendix Table 10

Reported estimates are marginal effects, which were multiplied by 100 in order to be interpreted as the percentage point difference in prevalence

95% confidence intervals are in parentheses

## Discussion

Our study found that longer paid maternity leave policy was associated with women’s increased role in economic decision making in the household and improved attitudes toward women’s right to work. In addition, we found that the egalitarian changes in attitudes were present in both women and men.

There are several possible mechanisms through which increased paid maternity leave policy may lead to these outcomes. First, paid leave, which grants time off with wage replacement, provides financial security to women and their families during maternity leave—prompting a shift from the traditional view of men as sole providers, encouraging people to value women’s financial contributions in new ways, and potentially creating a more supportive cultural context for women to work. Second, as better paid maternity leave policy is provided, women are increasingly encouraged to return to work and resume the role of a provider, countering traditional gendered expectations and thereby contributing to a shift away from restrictive norms that encourage discriminatory practices toward working mothers.

Paid maternity leave policies help provide financial stability to women and their families and support women’s labor force participation, outcomes that promote more equitable gender norms. This study’s findings are consistent with evidence from a longitudinal study of national changes in parental leave legislation, which found that policies that encourage fathers to take time off are associated with more egalitarian attitudes towards women's workforce participation [41]. Parental leave policies that facilitate women’s paid employment and incentivize men to share domestic labor can change attitudes about men’s and women’s roles in both the home and the workplace. These findings suggest that parental leave legislation can play an important role in advancing more egalitarian gender norms, which in turn serve as critical catalysts for improving a wide range of women’s and girls’ opportunities and outcomes.

Our findings in this study should be considered in light of some limitations. First, the parallel trends assumption is difficult to test. We lacked longitudinal measurements on the attitudinal measures for some sampled countries in the pre-intervention period. Second, although we included individual-level and country-level characteristics as covariates, as well as year and country fixed effects, uncontrolled time-varying confounding is still possible. Improved paid maternity leave policy may have been one component of a group of egalitarian legislative changes in some cases that increase gender equality by reducing the employment barriers and discrimination that women often face in the workplace. Third, because of the lack of information on policy compliance or enforcement, the intent-to-treat estimate obtained in our study may be downwardly biased.

Furthermore, although the dataset provides comparable information across countries for a sample of couples, it does not have detailed information about women’s participation in the labor market before having their last child, which would have allowed us to identify women in the formal labor market, the beneficiaries of maternity leave policies. As a result, an average population effect may underestimate the true effect of paid maternity leave when provided to all women because many women in the sample may not directly benefit from the policy change.

## Conclusion

Our findings expand on previous literature about the benefits of paid maternity leave policies. We found that more weeks of paid maternity leave available were positively related to more gender-equal norms regarding economic roles both in the household and workplace, measured through women’s and their partners’ or spouses’ perceptions about female participation in economic decision making and men’s and women’s attitudes toward equal access to jobs when jobs are scarce. Future research should consider the role of other policies in the reduction of social and economic gender inequalities, and the significance of gender norms as a mechanism for improving overall health, wellbeing and economic outcomes.

## Appendix

See Tables 9, 10, 11, 12, 13, 14

**Table 9:** Description of control variables included in the analyses of decision making in the household

Variable	Description
*Individual-level covariates*	
Women’s age	Continuous measure of women’s age. To consider a potential non-linear association, we also include age squared
Women’s education	Categorical measure of women’s education level: no education (= base category), primary, secondary, and higher
Women’s relationship to household’s head	Categorical variable indicating relationship to household head: head/wife/co-spouse (= base category), daughter (including adopted and foster), daughter-in-law, and other
Difference in age	Categorical variable: wife older than partner or spouse, same age (base category), partner or spouse is 1 or 2 years older, partner or spouse is 3 to 5 years older, partner or spouse is 6 to 10 years older, and partner or spouse is more than 10 years older
Difference in education	Categorical variable: wife’s education is higher than partner’s or spouse’s education, their education level is the same (base category), and partner’s or spouse’s education is higher than wife’s education
Number of children	Categorical variable indicating the number of children living in the household: one (base category), zero, two, three, and four or more
Area of residence	Indicator of rural residence constructed by the DHS
Household’s wealth index	Categorical variable constructed by the DHS: poorest (base category), poorer, middle, richer, and richest
Number of other women in the household	Categorical variable indicating the number of other women 15 years old or older in the household: none (base category), one, and two or more
*Country-level covariates*	
GDP per capita, survey year	Logarithm of GDP per capita of the year of the survey (constant 2011 international purchasing power parity dollars). Source: World Bank
Paid paternity leave, year prior to year of last child’s birth	Indicator of whether the country had paid leave reserved only for fathers the year prior to the women’s last childbirth. Source: PROSPERED and WORLD

**Table 10:** Description of control variables included in the analyses of gender norms at work

Variable	Description
*Individual-level covariates*	
Sex	Binary measure of respondent’s sex: male (= reference category) and female
Age	Continuous measure of respondent’s age
Year of birth	Continuous measure of respondent’s year of birth
Marital status	Categorical measure of respondent’s marital status: married or living together as married (= reference category), divorced/separated/widowed, and single/never married
Working status	Binary measure of respondent’s working status: not working (= reference category) and working
Education	Categorical measure of respondent’s education level: none or incomplete primary (= reference category), completed primary, incomplete secondary, complete secondary and some university or more
*Country-level covariates*	
GDP per capita, survey year	GDP per capita of the year of the survey (constant 2011 international purchasing power parity dollars). Source: World Bank
% female aged 15–64 participating in the labor force	Labor force participation rate, female (percentage of female population ages 15–64) (modeled ILO estimate). Source: World Bank
Unemployment female (% of female labor force)	Unemployment, female (% of female labor force) (modeled ILO estimate). Source: World Bank

**Table 11:** Effect of an increase in length of paid maternity leave policy on the odds of sole or joint decision-making authority in major household purchases for women, *N* = 100,294

	Women	Partners or spouses
	Odds ratios (95% CI)	Odds ratios (95% CI)
Panel A: Continuous measure of the duration of maternity leave		
*Exposure*		
1-month increase of paid maternity leave	1.40 (1.14, 1.70)	1.66 (1.36, 2.03)
*Individual-level covariates*		
Average women’s age	1.11 (1.08, 1.13)	1.05 (1.02, 1.07)
Women’s age squared	1.00 (1.00, 1.00)	1.00 (1.00, 1.00)
Women’s education		
No education	Ref	Ref
Incomplete primary	1.16 (1.09, 1.23)	1.20 (1.13, 1.28)
Complete primary	1.32 (1.22, 1.43)	1.37 (1.26, 1.49)
Incomplete secondary	1.76 (1.62, 1.91)	1.64 (1.50, 1.80)
Complete secondary	2.28 (2.01, 2.59)	1.71 (1.51, 1.94)
Higher	2.76 (2.36, 3.22)	2.57 (2.20, 3.01)
Women’s relationship to household’s head		
Head/wife/co-spouse	Ref	Ref
Daughter	0.89 (0.73, 1.07)	1.10 (0.91, 1.32)
Daughter-in-law	0.63 (0.56, 0.70)	0.58 (0.52, 0.65)
Other	0.96 (0.81, 1.13)	0.98 (0.83, 1.16)
Difference in age		
Wife is the same age as partner or spouse	Ref	Ref
Wife is older than partner or spouse	1.04 (0.91, 1.18)	0.97 (0.85, 1.10)
Partner or spouse is 1 or 2 years older	1.00 (0.89, 1.12)	1.05 (0.94, 1.18)
Partner or spouse is 3 to 5 years older	1.09 (0.98, 1.21)	1.08 (0.97, 1.21)
Partner or spouse is 6 to 10 years older	1.06 (0.95, 1.18)	1.11 (1.00, 1.24)
Partner or spouse is more than 10 years older	0.90 (0.81, 1.01)	1.05 (0.94, 1.18)
Difference in education		
Wife is at the same education level as partner or spouse	Ref	Ref
Wife’s education is higher than partner’s or spouse’s education	0.97 (0.91, 1.04)	0.87 (0.81, 0.93)
Partner’s or spouse’s education is higher than wife’s education	1.04 (1.00, 1.09)	1.10 (1.05, 1.16)
Number of children living in the household		
One	Ref	Ref
Zero	0.84 (0.67, 1.05)	1.12 (0.90, 1.39)
Two	0.95 (0.89, 1.02)	0.99 (0.92, 1.06)
Three	0.92 (0.85, 0.99)	0.97 (0.90, 1.05)
Four and more	0.89 (0.82, 0.96)	0.92 (0.85, 1.00)
Area of residence		
Urban	Ref	Ref
Rural	0.93 (0.87, 0.99)	0.94 (0.87, 1.01)
Household wealth index		
Poorest	Ref	Ref
Poorer	1.07 (1.00, 1.14)	1.01 (0.94, 1.08)
Middle	1.07 (1.01, 1.15)	1.04 (0.97, 1.12)
Richer	1.08 (1.01, 1.16)	1.13 (1.04, 1.23)
Richest	1.22 (1.11, 1.34)	1.24 (1.12, 1.37)
Number of other women in the household		
None	Ref	Ref
One	0.95 (0.90, 1.00)	0.92 (0.87, 0.98)
Two or more	0.85 (0.79, 0.91)	0.88 (0.81, 0.95)
*Country-level covariates*		
Mean (SD) Logarithm of GDP per capita, PPP (constant 2011 international $)	2.46 (1.53, 3.94)	1.74 (1.00, 3.03)
Availability of paid paternity leave, year prior to year of last child’s birth		
No	Ref	Ref
Yes	0.87 (0.78, 0.97)	1.04 (0.93, 1.16)
Panel B: Categorical measure of the duration of maternity leave (base = less than 8 weeks)		
*Exposure*		
0–7 weeks of paid maternity leave	Ref	Ref
8–13 weeks of paid maternity leave	1.47 (1.22, 1.77)	1.77 (1.46, 2.15)
14–30 weeks of paid maternity leave	1.67 (1.30, 2.16)	1.84 (1.42, 2.39)
*Individual-level covariates*		
Average women’s age	1.11 (1.08, 1.13)	1.05 (1.02, 1.07)
Women’s age squared	1.00 (1.00, 1.00)	1.00 (1.00, 1.00)
Women’s education		
No education	Ref	Ref
Incomplete primary	1.16 (1.09, 1.24)	1.20 (1.13, 1.28)
Complete primary	1.32 (1.22, 1.43)	1.37 (1.26, 1.49)
Incomplete secondary	1.76 (1.62, 1.91)	1.64 (1.50, 1.80)
Complete secondary	2.28 (2.01, 2.59)	1.71 (1.51, 1.94)
Higher	2.76 (2.36, 3.22)	2.58 (2.20, 3.02)
Women’s relationship to household’s head		
Head/wife/co-spouse	Ref	Ref
Daughter	0.89 (0.73, 1.07)	1.10 (0.91, 1.33)
Daughter-in-law	0.63 (0.56, 0.70)	0.59 (0.52, 0.66)
Other	0.96 (0.81, 1.13)	0.98 (0.83, 1.16)
Difference in age		
Wife is the same age as partner or spouse	Ref	Ref
Wife is older than partner or spouse	1.04 (0.91, 1.18)	0.97 (0.85, 1.10)
Partner or spouse is 1 or 2 years older	1.00 (0.89, 1.12)	1.05 (0.94, 1.18)
Partner or spouse is 3 to 5 years older	1.09 (0.98, 1.21)	1.08 (0.97, 1.21)
Partner or spouse is 6 to 10 years older	1.06 (0.95, 1.18)	1.11 (1.00, 1.24)
Partner or spouse is more than 10 years older	0.90 (0.81, 1.01)	1.05 (0.94, 1.18)
Difference in education		
Wife is at the same education level as partner or spouse	Ref	Ref
Wife’s education is higher than partner’s or spouse’s education	0.97 (0.91, 1.04)	0.87 (0.81, 0.93)
Partner’s or spouse’s education is higher than wife’s education	1.04 (1.00, 1.10)	1.11 (1.05, 1.16)
Number of children living in the household		
One	Ref	Ref
Zero	0.84 (0.67, 1.05)	1.12 (0.90, 1.39)
Two	0.95 (0.89, 1.02)	0.99 (0.92, 1.06)
Three	0.92 (0.85, 0.99)	0.97 (0.90, 1.05)
Four and more	0.89 (0.82, 0.96)	0.92 (0.85, 1.00)
Area of residence		
Urban	Ref	Ref
Rural	0.93 (0.86, 0.99)	0.93 (0.87, 1.01)
Household wealth index		
Poorest	Ref	Ref
Poorer	1.07 (1.00, 1.14)	1.01 (0.94, 1.08)
Middle	1.07 (1.01, 1.15)	1.04 (0.96, 1.12)
Richer	1.08 (1.00, 1.16)	1.13 (1.04, 1.22)
Richest	1.22 (1.11, 1.34)	1.23 (1.11, 1.37)
Number of other women in the household		
None	Ref	Ref
One	0.95 (0.90, 1.00)	0.92 (0.87, 0.98)
Two or more	0.85 (0.79, 0.91)	0.88 (0.81, 0.95)
*Country-level covariates*		
Mean (SD) Logarithm of GDP per capita, PPP (constant 2011 international $)	2.44 (1.53, 3.91)	1.70 (0.98, 2.95)
Availability of paid paternity leave, year prior to year of last child’s birth		
No	Ref	Ref
Yes	0.84 (0.76, 0.94)	1.03 (0.93, 1.15)

All regressions are weighted and adjusted for country, the last child’s year of birth, and survey year fixed effects

**Table 12:** Effect of a 1-month increase in length of paid maternity leave policy on the prevalence of individuals disagreeing with the statement “when jobs are scarce, men should have more right to a job than women”, in lower middle and low income countries, *N* = 53,811

	Disagree	Neither
*Exposure*		
1-month increase in length of paid maternity leave policy	41.5 (20.3, 62.6)	21.2 (8.2, 34.2)
*Individual-level covariates*		
Sex		
Male	Ref	Ref
Female	14.8 (12.2, 17.4)	1.1 (−0.4, 2.7)
Age	0.2 (−0.2, 0.6)	0 (−0.3, 0.2)
Year of birth	0.2 (−0.2, 0.6)	0 (−0.3, 0.2)
Marital status		
Married or living together as married	Ref	Ref
Divorced/separated/widowed	1.5 (−0.5, 3.6)	0.7 (−0.6, 1.9)
Single/never married	3.9 (1.9, 6.0)	0.6 (−0.4, 1.7)
Working status		
Not working	Ref	Ref
Working	3.0 (1.7, 4.2)	−0.1 (−1.2, 1.0)
Education		
None or incomplete primary	Ref	Ref
Completed primary	2.7 (1.2, 4.2)	−1.4 (−3.0, 0.2)
Incomplete secondary	4.5 (2.2, 6.7)	−2.0 (−4.2, 0.2)
Completed secondary	6.4 (4.4, 8.5)	−1.9 (−4.2, 0.4)
Some university or more	13.2 (10.5, 16.0)	−0.9 (−3.5, 1.8)
*Country-level covariates*		
GDP per capita	0 (0, 0)	0 (0, 0)
% Female aged 15–64 participating in the labor force	0.3 (−0.9, 1.6)	−1.2 (−1.7, −0.6)
Unemployment female (% of female labor force)	−0.7 (−1.1, -0.3)	0.8 (0.4, 1.2)

**Table 13:** Effect of a 1-month increase in length of paid maternity leave policy on the prevalence of individuals disagreeing with the statement “when jobs are scarce, men should have more right to a job than women”, in lower middle and low income countries, men only, *N* = 25,877

	Disagree	Neither
*Exposure*		
1-month increase in length of paid maternity leave policy	28.4 (8.1, 48.8)	24.7 (13.8, 35.5)
*Individual-level covariates*		
Age	0.3 (−0.2, 0.8)	−0.1 (−0.3, 0.2)
Year of birth	0.3 (−0.2, 0.7)	−0.1 (−0.3, 0.1)
Marital status		
Married or living together as married	Ref	Ref
Divorced/separated/widowed	−4.0 (−8.6, 0.6)	1.7 (−1.3, 4.7)
Single/never married	1.3 (−1.1, 3.7)	1.0 (−0.3, 2.3)
Working status		
Not working	Ref	Ref
Working	0.4 (−0.9, 1.7)	0.6 (−0.4, 1.5)
Education		
None or incomplete primary	Ref	Ref
Completed primary	1.9 (−1.7, 5.6)	−0.9 (−2.9, 1.1)
Incomplete secondary	3.3 (0.3, 6.4)	−0.4 (−2.9, 2.1)
Completed secondary	5.4 (2.1, 8.6)	−1.0 (−3.2, 1.2)
Some university or more	10.6 (7.1, 14.1)	1.1 (−1.5, 3.7)
*Country-level covariates*		
GDP per capita	0 (0, 0)	0 (0, 0)
% Females aged 15–64 participating in the labor force	0.4 (−0.8, 1.6)	−1.3 (−1.8, −0.8)
Unemployment female (% of female labor force)	−0.8 (−1.2, −0.5)	0.9 (0.6, 1.3)

**Table 14:** Effect of a 1-month increase in length of paid maternity leave policy on the prevalence of individuals disagreeing with the statement “when jobs are scarce, men should have more right to a job than women”, in lower middle and low income countries, women only, *N* = 27,934

	Disagree	Neither
*Exposure*		
1-month increase in length of paid maternity leave policy	54.1 (31.7, 76.5)	18.3 (2.8, 33.8)
*Individual-level covariates*		
Age	0.1 (−0.6, 0.8)	0.1 (−0.3, 0.4)
Year of birth	0.3 (−0.5, 1.0)	0.1 (−0.3, 0.4)
Marital status		
**Married** or living together as married	Ref	Ref
**Divorced**/separated/widowed	4.0 (2.1, 6.0)	0.4 (−0.7, 1.5)
**Single**/never married	6.4 (3.6, 9.3)	0.7 (−1.2, 2.7)
Working status		
**Not** working	Ref	Ref
**Working**	3.5 (1.6, 5.4)	−0.1 (−1.5, 1.3)
Education		
**None** or incomplete primary	Ref	Ref
**Completed** primary	3.1 (0.8, 5.4)	−1.3 (−3.3, 0.6)
**Incomplete** secondary	5.1 (2.1, 8.0)	−3.0 (−5.2, −0.8)
**Completed** secondary	7.1 (4.6, 9.6)	−2.1 (−4.7, 0.6)
**Some** university or more	15.2 (11.8, 18.6)	−1.9 (−4.8, 0.9)
*Country-level covariates*		
GDP per capita	0 (0, 0)	−0 (−0, −0)
% Females aged 15–64 participating in the labor force	0.2 (−1.1, 1.5)	−1.0 (−1.7, −0.3)
Unemployment female (% of female labor force)	−0.6 (−1.0, −0.1)	0.7 (0.3, 1.1)

## Abbreviations

DHS: Demographic and health surveys
GDP: Gross domestic product
LMICS: Low- and middle-income countries
PROSPERED: Policy-relevant observational studies for population health equity and responsible development
PPP: Purchasing power parity
SD: Standard deviation
UN: United nations
WVS: World values survey

## Declarations

### Availability of Data and Material

Longitudinal data measuring national maternity leave policies for each United Nations (UN) member state were made available through McGill University’s Policy-Relevant Observational Studies for Population Health Equity and Responsible Development (PROSPERED) project and the WORLD Policy Analysis Center.

### Code Availability

Statistical analyses were performed using Stata software version 16 (Stata Corp, College Station, TX).
